# Adaptive wavelet base selection for deep learning-based ECG diagnosis: A reinforcement learning approach

**DOI:** 10.1371/journal.pone.0318070

**Published:** 2025-02-03

**Authors:** Qiao Xiao, Chaofeng Wang

**Affiliations:** 1 School of Computer Science, University of South China, Hengyang, Hunan, China; 2 Department of Community Health, Faculty of Medicine and Health Sciences, Universiti Putra Malaysia, Serdang, Selangor, Malaysia; George Emil Palade University of Medicine PharmacyScience and Technology of Targu Mures: Universitatea de Medicina FarmacieStiinte si Tehnologie George Emil Palade din Targu Mures, ROMANIA

## Abstract

Electrocardiogram (ECG) signals are crucial in diagnosing cardiovascular diseases (CVDs). While wavelet-based feature extraction has demonstrated effectiveness in deep learning (DL)-based ECG diagnosis, selecting the optimal wavelet base poses a significant challenge, as it directly influences feature quality and diagnostic accuracy. Traditional methods typically rely on fixed wavelet bases chosen heuristically or through trial-and-error, which can fail to cover the distinct characteristics of individual ECG signals, leading to suboptimal performance. To address this limitation, we propose a reinforcement learning-based wavelet base selection (RLWBS) framework that dynamically customizes the wavelet base for each ECG signal. In this framework, a reinforcement learning (RL) agent iteratively optimizes its wavelet base selection (WBS) strategy based on successive feedback of classification performance, aiming to achieve progressively optimized feature extraction. Experiments conducted on the clinically collected PTB-XL dataset for ECG abnormality classification show that the proposed RLWBS framework could obtain more detailed time-frequency representation of ECG signals, yielding enhanced diagnostic performance compared to traditional WBS approaches.

## Introduction

According to a report issued by the American Heart Association [1], cardiovascular diseases (CVDs) emerge as the leading cause of mortality worldwide, with the number of individuals affected by CVDs projected to rise to 23.6 million by 2030 [2]. The ECG is a critical physiological recording obtained via electrodes placed on the body surface that measures the heart’s electrical activity. It provides essential diagnostic information for detecting CVDs. ECG signals can reveal symptoms of heart-related pathologies, which are crucial for the prompt diagnosis and effective monitoring of CVDs. They also have the potential to facilitate rapid medical interventions for patients [3]. Therefore, the precise and efficient diagnosis of ECG signals for identifying heart diseases is instrumental in ensuring timely treatment and early intervention, which could dramatically decrease mortality rates associated with CVDs [4].

However, manual ECG diagnosis requires specialized knowledge and significant timenvestment from physicians, consuming substantial medical resources and potentially causing diagnostic backlog. Consequently, there is a growing demand for automated ECG classification technologies to address the increasing burden of CVDs [5]. To accelerate the automation of the ECG diagnostic process for clinical applications, many existing studies employ DL to directly map ECG signals to their corresponding categories [6–8]. In addition, feature engineering which extract significant features from ECG signals could help further improve the classification performance and efficiency of DL-based ECG classification [9]. Among various feature extraction methods, the wavelet transform (WT) is particularly effective for extracting time-frequency information of signals and could reveal frequency variations over time, which is suitable to obtained refined features from non-stationary signals like ECGs [10].

**Fig 1:**
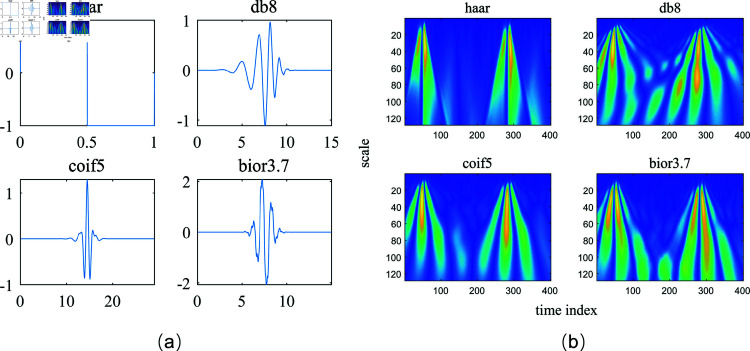
An illustration of different wavelet bases and their corresponding wavelet features obtained from the same ECG signal. (a) Different wavelet bases. (b) Wavelet features obtained with different bases.

The WT is widely used in DL-based ECG classification [11] as it could utilize wavelet base functions to filter and decompose ECG signals into different sub-bands across various time scales [12]. For instance, [13] utilizes continuous wavelet transform (CWT) to convert ECG signals into the time-frequency domain and employ convolutional neural networks (CNNs) to extract features from the time-frequency maps. It achieved an improvement in the F1 score by 4.75% to 16.85% compared to competing methods without WD for arrhythmia classification. In [14], 24 wavelet features are severed as the input to a multi-layer perceptron (MLP) neural network, and a classification accuracy of 96.5% can be achieved for arrhythmia detection. [15] proposes a novel deep bidirectional LSTM network that takes wavelet sequences at each decomposition level as input features for ECG classification, resulting an accuracy of 99.39% on the MIT-BIH Arrhythmia Database. DL-based ECG classification with wavelet features maintains classification performance while benefiting from lower model complexity, making it practical for applications with limited computational and storage resources [16]. Selecting an appropriate wavelet base function is crucial for accurately capturing the characteristics of time-varying signals, as shown in Fig 1. It can be seen that the detailed time-frequency characteristics captured differ depending on the chosen wavelet base. Hence, the choice of wavelet base function is a critical pre-determined parameter for WT in DL-based ECG classification [9]. Despite the powerful capabilities of WT for feature extraction, the selection of an optimal wavelet base remains a complex and uncertain challenge [10].

To select appropriate wavelet bases, many existing studies pre-select the base based on expert experience [17]. Some studies consider selecting optimal wavelet base based on the correlation or similarity between the wavelet base and the signals to be analyzed. For instance, [18] determine the optimal wavelet base function for ECG signal denoising by calculating the correlation coefficients between the ECG signal and different wavelet base functions. The basis with the highest correlation coefficient is considered as optimal. Recently, selecting the optimal wavelet bases based on the performance of the targeted application becomes popular. [19] proposes a cross-validation approach to select wavelet bases, where the wavelet combination that yields the highest detection performance during the validation is utilized for further analysis. [20] conducts a thorough quantitative analysis to evaluate the denoising performance of 115 potential wavelet base functions (from 6 wavelet families). The optimal wavelet base can be determined based on the signal-to-noise ratio (SNR) after denoising. In[21], the wavelet base which yields the highest denoising performance and arrhythmia classification performance simultaneously is considered as optimal. These wavelet parameter selection methods indeed could find an optimal wavelet base appropriate for the characteristics of most ECG signals, resulting in higher average performance. However, using a single wavelet base for all ECG signals may overlook signals that deviate from the majority, as ECG signals in different categories, especially abnormal ones, exhibit significant variations in the time-frequency domain.

In this study, we focus on a dynamic approach to determine wavelet bases for ECG signals to enhance DL-based ECG diagnosis. By selecting wavelet bases which coincides with the unique characteristics of each ECG signal, we aim to generate wavelet features that are more detailed and distinguishable. Leveraging RL [22], the wavelet base selection (WBS) process is modeled as a stateless Markov Decision Process (MDP) [23]. Here, an RL agent is trained to optimize its action, i.e., selecting wavelet bases, to maximize the reward induced by more appropriate selection of wavelet bases. In our previous study [24], RL was successfully adopted for selection of the optimal parameters for short-time Fourier transform (STFT), which inspires us to apply RL for adaptively select wavelet bases for DL-based ECG classification.

Our main contributions are as follows:

- This is the first study to systematically consider selecting wavelet bases for signals within an RL framework, where an agent is trained to choose wavelet bases to obtain improved wavelet features, thereby immediately enhancing classification performance.
- The wavelet base is customized for each individual ECG signal, allowing for the capture of the most significant wavelet features relevant to its respective category.
- The efficacy of the proposed approach is validated in ECG abnormality classification through the clinically collected PTB-XL ECG database compared to competing methods.

## Materials and methods

**Fig 2:**
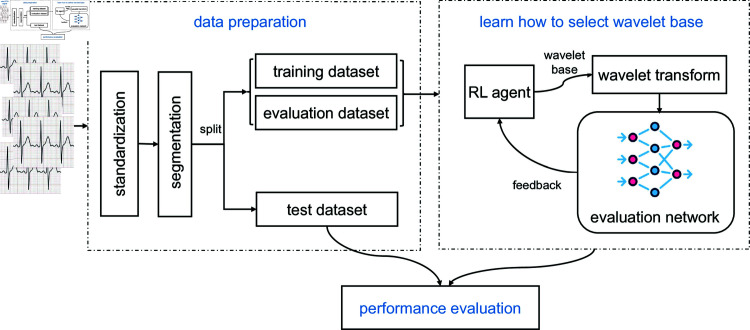
The overall workflow of this study.

The overall workflow of the proposed approach is illustrated in Fig 2. First, raw ECG signals undergo preprocessing to standardize the data by scaling inputs and segmenting the signals for label aggregation. The preprocessed signals are then split into training, validation, and test datasets. An RL agent is trained to adaptively select appropriate wavelet bases for the ECG signals in the training dataset. Simultaneously, the agent’s selection strategy is guided through feedback from an evaluation network, which continuously assesses the diagnostic performance obtained with the continuous wavelet transform (CWT) of ECG signals in the evaluation dataset, using the wavelet bases provided by the agent. Finally, the trained RL agent is evaluated on the test dataset to assess the effectiveness of its WBS strategy.

### Continuous wavelet transform

In this study, the CWT is employed to extract time-frequency features from ECG signals as it could extract detailed characteristics of ECG signals at adjustable time-frequency resolution. Consider a real-valued signal *x* ( *t* ) , the CWT of *x* ( *t* )  by using a wavelet base *ψ* ( *t* )  can be formulated as


$$\begin{array}{c} W(c,b)=\frac{1}{\sqrt{c}}\int_{-\infty }^{+\infty }x(t)\psi (\frac{t-b}{c})dt \end{array}$$ (1)

where *c* and *b* correspond to the parameters of scale and time shift, respectively, *W* ( *c* , *b* )  represents the wavelet coefficient at scale *c* and the time shift *b*, the term $\psi (\frac{t-b}{c})$ describes the wavelet base *ψ* ( *t* )  under the translational and scaling transformations. The wavelet coefficient *W* ( *c* , *b* )  essentially quantifies the similarity between signal and the wavelet function $\psi (\frac{t-b}{c})$, producing features across multiple temporal regions and frequencies. This enables the capture of time-frequency characteristics in the signal at varying resolutions.

Denote $c=[c_{1},c_{2},\cdots \;,c_{M}]$ and $b=[b_{1},b_{2},\cdots \;,b_{N}]$ as the vectors containing sampled values in the scale and shift domains, respectively. The sampled version of the continuous wavelet transform (CWT) in the time-frequency domain can be represented as


$$\begin{array}{c} W={\left\{W(c_{i},b_{i})\right\}}_{\left(i,j)\in [c_{1},c_{2},\cdots \;,c_{M}]\times [b_{1},b_{2},\cdots \;,b_{N}\right]} \end{array}$$ (2)

where $W(c_{i},b_{i})$ is the  ( *i* , *j* ) th element in *W*. The matrix *W* represents the discretized CWT results, capturing the time-frequency characteristics of the signal. These features, represented by *W*, can then be regarded as wavelet-derived features suitable for input into DL models for further analysis and classification.

The wavelet bases exhibit variable time-frequency characteristics, as shown in Fig 1. Commonly used wavelet bases in signal analysis include the Haar wavelet, Daubechies wavelet (dbN), Symlet wavelet (symN), Coiflet wavelet (coifN), and Biorthogonal wavelet (biorNr.Nd) [25]. The Haar wavelet is the simplest to compute but is discontinuous in the time domain. Daubechies wavelets, with their extreme phase and higher vanishing moments, are suitable for reconstructing smooth signals but are more computationally complex and asymmetric. Symlet wavelets improve upon Daubechies wavelets by offering better symmetry and reduced phase distortion. Coiflet wavelets provide high symmetry and effective frequency band partitioning. Biorthogonal wavelets introduce biorthogonality, resolving the conflict between symmetry and precise signal reconstruction.

The wavelet features obtained from the same ECG signal using different wavelet bases through CWT can vary as their time-frequency characteristics change depending on the selected wavelet base, highlighting the importance of choosing the right one. An appropriate wavelet base is crucial for extracting relevant features from ECG signals that are indicative of different diagnostic categories. This study aims to develop a systematic method for selecting the optimal wavelet base for individual ECG signals, enhancing the feature extraction capabilities of CWT and thereby improving the classification accuracy of models in distinguishing between various ECG categories.

### RL basics

**Fig 3:**
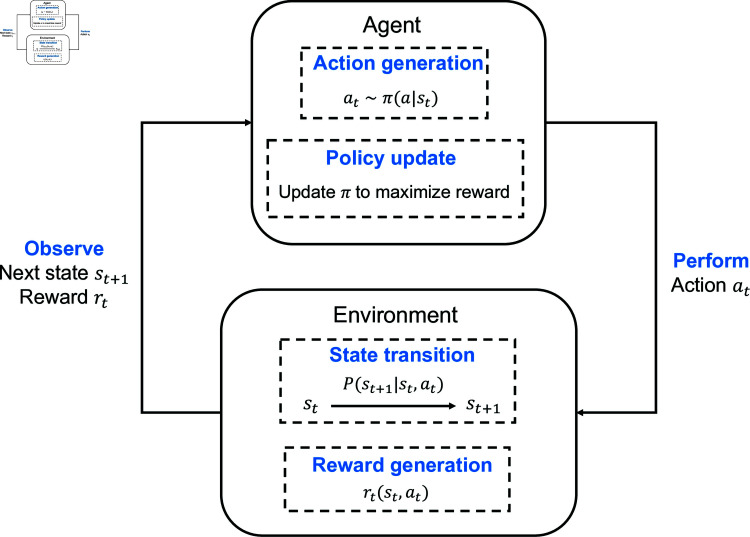
Structure of reinforcement learning.

RL is an approach that involves learning to generate actions to maximize cumulative rewards through interaction with an environment. The structure of RL is illustrated in Fig 3. At its core, an agent interacts with the environment by taking actions based on the current system state and receiving rewards in return. The agent aims to learn the optimal action at each time step to maximize the long-term cumulative reward through continuous learning and policy improvement. The problem addressed by RL can be modeled as a MDP [26], characterized by the tuple  { *S* , *A* , *P* , *R* , *γ* } , where:

- *S* is the state space, representing the set of all possible system states.
- *A* is the action space, representing the set of all possible actions.
- *P* is the state transition probability, representing the probability distribution of transitioning from one state to another. Specifically, $P(s^{\prime }|s,a)$ denotes the probability of transitioning to state $s^{\prime }$ from state *s* after taking action *a*.
- *r* is the reward function, representing the immediate reward received after performing an action in a given state, denoted as *R* ( *s* , *a* ) .
- *γ* is the discount factor, which determines the present value of future rewards and lies within the interval  [ 0 , 1 ] .

In an MDP, the policy *π* is a mapping function that specifies the action *a* to be taken in each state *s*, i.e., *π* : *S* → *A*. The goal of reinforcement learning is to find an optimal policy $\pi^{*}$ that maximizes the expected cumulative reward. Specifically, the objective can be expressed as maximizing the following expected discounted sum:


π*= arg ⁡ max ⁡ πE [∑t=0∞γtR(st,at)∣s0=s] (3)

where $a_{t}\sim \pi (a|s_{t})$ is the action given by a policy maker *π* at state $s_{t}$, $s_{t+1}\sim P(s|s_{t},a_{t})$ denotes the transition probability of state $s_{t+1}$ given the current state $s_{t}$ and action $a_{t}$, and *γ* is the discount factor used to balance short-term and long-term rewards.

In this study, an agent is employed that follows a policy, taking the ECG signal as input and generating an action that selects the optimal wavelet base for the CWT of the ECG signals. the agent continuously refines its selection strategy, improving the accuracy of the ECG classification task over time.

### RL-based WBS

To address the problem of selecting the appropriate wavelet base for ECG diagnosis, we propose the RL-based wavelet base selection (RLWBS) framework. This approach systematically determines the optimal wavelet base for each ECG signal, enhancing feature extraction and improving classification performance of ECG signals. The WBS problem is formulated as a MDP within an RL framework, where an RL agent interacts with a specially designed environment to iteratively learn and refine the rationale of WBS based on the classification feedback.

#### RLWBS framework.

**Fig 4:**
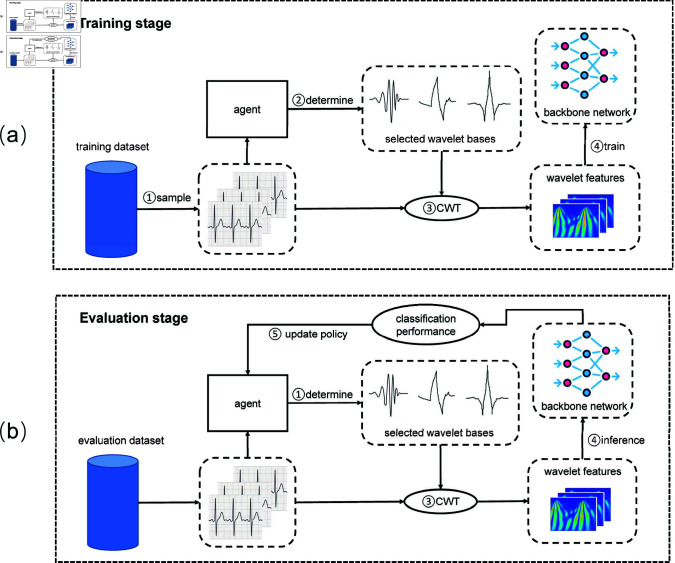
An illustration of the training and evaluation stages. (a) Training stage. (b) Evaluation stage.

In this study, as a data-driven method, the ECG data to train the policy maker is divided into training and evaluation datasets, i.e., *T* and *V*, respectively. Then, the WBS process can be further divided into two stages as illustrated in Fig 4.

In the training stage, as shown in Fig 4, at the beginning of its *t*th learning iteration, a mini-batch of ECG signals is randomly sampled from the training dataset *T* and grouped as $X_{t}$, i.e.,


$$\begin{array}{c} X_{t}=\{x_{1t},x_{2t},\cdots \;,x_{Bt}\} \end{array}$$ (4)

where $x_{it}$ is the *i*th ECG signal in a mini-batch of *B* ECG signals in the *t*th iteration.

Denote the action space $A=\{a^{\left(1\right)},a^{\left(2\right)},\cdots \;,a^{\left(N_{a}\right)}\}$ where $a^{\left(j\right)}$ is the *j*th candidate action and $N_{a}$ is the total number of candidate actions. Let $\pi_{\theta }(x)$ represent the policy network, which takes an ECG signal *x* as input and outputs a probability vector


$$\begin{array}{c} P(a|x)=[p(a=a^{\left(1\right)}|x),p(a=a^{\left(2\right)}|x),\cdots \;,p(a=a^{\left(N_{a}\right)}|x)] \end{array}$$ (5)

where *P* ( *a* | *x* )  corresponds to the probability distribution for selecting an action *a* conditioned on *x*, with $p(a=a^{\left(j\right)}|x)$ indicating the conditional probability of choosing the *j*th action (i.e., the *j*th wavelet base) on *x*. The action for the ECG signal $x_{it}$, denoted as $a_{it}$, is determined by sampling from the probability distribution $P(a|x_{it})$, i.e.,


$$\begin{array}{c} a_{it}\sim P(a|x_{it}). \end{array}$$ (6)

We aggregate all the actions from *V* as the action for the whole system as


$$\begin{array}{c} a_{t}:=[a_{1t},a_{2t},\cdots \;,a_{Bt}] \end{array}$$ (7)

Once $a_{it}$ (the chosen wavelet base) is selected, the wavelet features for the ECG signal $x_{it}$ are extracted using the CWT with the selected wavelet base, denoted as $W_{it}$. The resulting wavelet features, $W_{t}=\{W_{1t},W_{2t},\cdots \;,W_{Bt}\}$, along with their respective categories, are used to train a backbone neural network *f*. This trained network $f_{t}$ in the *t*th iteration serves as the evaluation network, assessing classification accuracy and generating a reward. This reward evaluates the effectiveness in WBS of the policy network, guiding further refinement of its strategy.

In the evaluation stage, as illustrated in Fig 4, each ECG signal in the evaluation dataset, i.e., $x_{i}^{\left(v\right)}\in V$, is input to the policy network $\pi_{\theta }$ to obtain its corresponding action selection probabilities $P(a|x_{i}^{\left(v\right)})$. Unlike in the training stage, the action for each signal is selected with the highest output probability from the policy network, i.e.,


ai(v)= arg ⁡ max ⁡ aP(a|xi(v)) (8)

where $a_{i}^{\left(v\right)}$ is the selected action, i.e., the wavelet base index, for the signal $x_{i}^{\left(v\right)}$. The wavelet features $W_{v}$ for the ECG signals in the evaluation dataset are then obtained based on the selected actions ${\left\{a_{i}^{\left(v\right)}\right\}}_{i=1}^{\left|V\right|}$ where  | *V* |  denotes the size of the evaluation dataset. The backbone model, $f_{t}$, trained in the previous stage, serves as the evaluation network, providing classification performance using the wavelet features $W_{v}$. The inference accuracy on the evaluation dataset, denoted as $\eta_{t}$, reflects the effectiveness of the wavelet features $W_{t}$ generated by the policy network $\pi_{\theta }$ in training the backbone network *f*. Hence, Thus, the system state in this study is defined as


$$\begin{array}{c} s_{t}:=\{X_{t},\pi_{\theta }\}. \end{array}$$ (9)

Finally, reward in the *t*th iteration can be described as


$$\begin{array}{c} r_{t}(s_{t},a_{t}):=\eta_{t}(X_{t},\pi_{\theta },f_{t},V) \end{array}$$ (10)

Here, $\eta_{t}$ is influenced by the performance of the evaluation network $f_{t}$, which is trained on $X_{t}$ with wavelet bases selected by $\pi_{\theta }$, and further guided by the classification accuracy calculated on the evaluation dataset.

The policy network $\pi_{\theta }$ then adjusts its weights *θ* to generate improved actions, aiming to maximize the expected reward. This optimization is achieved using the policy gradient method [27], which allows the network to refine its WBS strategy over time. The detailed process of this adjustment using policy gradients will be described in the following subsection.

In this study, the candidate wavelet bases include the Haar, Daubechies, Biorthogonal, Coiflets, and Symlets wavelet families, as shown in Table 1. These wavelet families are selected based on a comprehensive evaluation of their properties, including compact support, orthogonality, and vanishing moments, which are crucial for effective feature extraction and classification performance [28]. Hence, the action set for each ECG signal can be defined as $A=\{1,2,\cdots \;,i,\cdots \;,N_{a}\}$, where *i* corresponds to the index of the wavelet base listed in Table 1. Each index represents a specific wavelet base that the RL agent can select for feature extraction.

**Table 1:** The candidate wavelet bases that can selected by the RL agent. The wavelet base is indexed, and the number in parentheses is subsequently used to represent the corresponding wavelet base.

Wavelet family	Wavelet bases (Index)
Haar	haar (1)
Daubechies	db2 to db20 (2 to 20)
Symlets	sym3 to sym8 (21 to 26), sym10 (27), sym20 (28)
Coiflets	coif3 to coif5 (29 to 31)
Biorthogonal	bior1.1(32), bior2.2(33), bior3.3(34), bior4.4(35)

#### Update of policy network.

In this study, the policy gradient (PG) algorithm [27] is employed to optimize the policy, aiming to maximize the probability of selecting the optimal action given a state [29]. We define the policy $\pi_{\theta }(a|s_{t})$ as:


$$\begin{array}{c} \pi_{\theta }(a|s_{t}):=\pi_{\theta }(a|x_{1t})\cdot \pi_{\theta }(a|x_{2t})\cdots \pi_{\theta }(a|x_{Bt}). \end{array}$$ (11)

This represents the joint distribution of action selection for all ECG signals in the state $s_{t}$, where *a* contains the corresponding actions for the signals in the state. Each $\pi_{\theta }(a|x_{it})$ is the probability of selecting action *a* (i.e., the wavelet base) for the *i*th ECG signal $x_{it}$ within the mini-batch.

According to the PG algorithm, the gradient of the sampled version of the expected cumulative reward with respect to the network weights *θ* based on collected state-action pairs and rewards can be calculated as


∇ ⁡J(θ)= [∑t=0T∇ ⁡θ log ⁡ πθ(at∣st)∑k=tTγk-trk], (12)

where ∇ ⁡θ log ⁡ πθ(at∣st) represents the gradient of the log-probability of selecting the action $a_{t}$ given state $s_{t}$ under the current policy network $\pi_{\theta }$. According to (5), (6), and (11), the gradient *∇* ⁡ *J* ( *θ* )  can be further expressed as


∇ ⁡J(θ)= [∑t=0T ∑i=1B∇ ⁡θ log ⁡ p(a=ait∣xit)∑k=tTγk-trk] (13)

Finally, based on the gradient descent theorem, the learnable weights of the policy network *θ* can be updated by performing gradient ascent:


$$\begin{array}{c} \theta \leftarrow \theta +\alpha \nabla J(\theta ), \end{array}$$ (14)

where *α* is the learning rate for the weights of the policy network.

**Fig 5:**
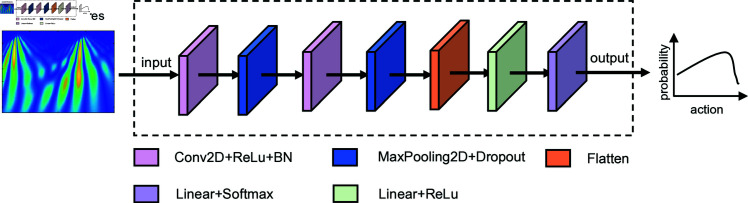
Structure of the policy network.

The detailed design of the policy network is shown in Fig 5. The input signal first passes through two modules consecutively, each containing Conv2D, ReLU, and BN operations, followed by MaxPooling2D and Dropout. The corresponding output from the two modules is then flattened and passed through a linear layer with a ReLU activation function. Finally, two branches, each including a linear layer and a softmax layer, are utilized to generate the two elements in the action individually. The final output represents the probability distributions of selecting the wavelet base.

By continuously updating the policy network using the PG algorithm, the model improves its ability to select the most effective wavelet bases for the corresponding ECG signals to exhibit better features for ECG classification.

#### Evaluation network.

To evaluate the effectiveness of the actions generated by the policy network $\pi_{\theta }$ and guide its learning of more appropriate wavelet bases, a deep neural network *f* is employed as the critic within the RL framework [30].

During the *t*th iteration of the training stage, the network *f* is trained as the backbone using the wavelet features $W_{t}$. The training continues until the backbone achieves a sufficient level of accuracy, at which point its generalization capability reflects the appropriateness of the actions $a_{t}$ generated by the policy network $\pi_{\theta }$.

To further assess the actions $a_{t}$ chosen by the policy network, the prediction accuracy $\eta_{t}$ is measured by inputting the wavelet features $W_{v}$ from the evaluation dataset into the trained backbone network *f*. These wavelet features are derived from the wavelet bases corresponding to the actions ${\left\{a_{i}^{\left(v\right)}\right\}}_{i=1}^{\left|V\right|}$ selected by the policy network. This evaluation effectively quantifies the capability of WBS mechanism of the policy network.

The accuracy $\eta_{t}$ serves as the reward, providing a direct assessment of the effectiveness of the wavelet bases $a_{t}$ selected by the policy network. At the end of each learning iteration, the weights of the backbone network *f* are reset to their initial values, ensuring that training begins from a fresh state at the start of each iteration.

**Algorithm 1**: RLWBS in the *t*th learning iteration

1:  Sample a mini-batch of ECG signals from the training dataset, denoted as $s_{t}$

2:  Select actions for each ECG signal $x_{it}$ in $s_{t}$ using the policy network $\pi_{\theta }$, i.e., $a_{it}\sim \pi_{\theta }(a|x_{it})$

3:  Perform CWT to extract wavelet features $W_{t}$ from the signals in $s_{t}$ based on the selected actions $a_{t}$

4:  Train the backbone network *f* using the wavelet features $W_{t}$ and their corresponding labels

5:  Apply the policy network to the evaluation dataset, obtain the wavelet bases for the ECG signals, and compute their corresponding wavelet features $W_{v}$

6:  Evaluate the prediction accuracy $\eta_{t}$ on the evaluation dataset using the trained network *f*, and use it as the reward $r_{t}$

7:  Update the policy network $\pi_{\theta }$ by performing gradient ascent to adjust its parameters *θ*, using Eqs. (13) and (14)

8:  Reset the backbone network *f* to its initial state

Algorithm 1 describes the proposed RLWBS framework in the *t*th learning iteration. The process continues until the inference accuracy on the evaluation dataset shows only minor variations. At this point, the entire process terminates, and the policy network can be applied to ECG signals for the targeted applications.

### Performance metrics

The classification performance of the proposed approach is evaluated using the metrics of precision, recall, sensitivity, specificity, Area Under the Curve (AUC), F1, and Matthews Correlation Coefficient (MCC), which are defined as:


$$\begin{array}{c} \text{Precision}=\frac{\text{TP}}{\text{TP}+\text{FP}}, \end{array}$$ (15)

$$\begin{array}{c} \text{Recall}=\frac{\text{TP}}{\text{TP}+\text{FN}}, \end{array}$$ (16)

$$\begin{array}{c} \text{Sensitivity}=\frac{\text{TP}}{\text{TP}+\text{FN}}, \end{array}$$ (17)

$$\begin{array}{c} \text{Specificity}=\frac{\text{TN}}{\text{TN}+\text{FP}}, \end{array}$$ (18)

$$\begin{array}{c} \text{AUC}=\frac{\text{Sensitivity}}{\text{Specificity}}, \end{array}$$ (19)

$$\begin{array}{c} F1=2\times \frac{\text{Precision}\times \text{Recall}}{\text{Precision}+\text{Recall}}, \end{array}$$ (20)

$$\begin{array}{c} MCC=\frac{\text{TP}\times \text{TN}-\text{FP}\times \text{FN}}{\sqrt{\left(\text{TP}+\text{FP})(\text{TP}+\text{FN})(\text{TN}+\text{FP})(\text{TN}+\text{FN}\right)}}, \end{array}$$ (21)

where TP, TN, FP and FN represent the true positive , true negative, false positive, and false negative predicted values, respectively.

Additionally, to calculate the macro-averaged metrics, the corresponding metrics are first computed individually for each category. These values are then averaged, assigning equal weight to each category irrespective of its sample size, to derive the final macro-averaged metrics.

## Results and discussion

In this section, we assess the effectiveness of the proposed RLWBS framework for autonomously selecting optimal wavelet bases. The proposed method is implemented in Python using the PyTorch framework. For this evaluation, we perform experiments on the publicly available PTB-XL ECG database [31]. Initially released in 2020, the dataset includes 21,799 clinical 12-lead ECG recordings from 18,869 patients, each lasting 10 seconds. This study focuses on multi-label classification across five superclass categories: normal ECG (NORM), conduction disturbance (CD), hypertrophy (HYP), myocardial infarction (MI), and ST/T change (STTC). Given that a single ECG can carry multiple labels, this creates a multi-label classification scenario. The PTB-XL dataset adheres to the inter-patient paradigm [32], ensuring that records from the same patient appear exclusively in either the training or test sets, with no overlap.

For baseline comparisons, we include a commonly used approach that selects the wavelet base achieving the highest classification accuracy in N-fold cross-validation, as in [33] (referred to here as CV-WBS). Additionally, we evaluate an energy and Shannon entropy-based method (referred to as EE-WBS) as used in [34], which selects the optimal wavelet base based on the energy-to-Shannon entropy ratio.

### Performance improvement with RLWBS

We first evaluate the proposed RLWBS framework on five DL models used as benchmarkfor ECG classification on the PTB-XL dataset as listed in [35], i.e., XResNet [36], Inception [37], ResNet [38], LSTM [39], and LSTM-bidir [39]. Additionally, we include the state-of-the-art model LDM-XResNet, which has demonstrated the highest classification performance in [40]. Originally developed for 1D ECG inputs, these models have been adapted to process 2D wavelet features in this study, including modifications such as substituting 1D convolutional layers with 2D convolutional layers to enable compatibility with 2D feature inputs.

Table 2 presents the macro-AUC and macro-F1 scores of the tested models on the PTB-XL dataset, comparing results across different wavelet selection methods. Models paired with the proposed RLWBS method consistently achieved higher macro-AUC and macro-F1 scores than those using CV-WBS and EE-WBS, demonstrating the efficacy of the proposed RLWBS. This result suggests that RLWBS generates more informative wavelet features, enhancing the ability of model to differentiate between categories. In subsequent analyses, LDM-XResNet is selected as the classifier to further evaluate different WBS approaches.

**Table 2:** Performance comparison of various DL models using different WBS methods.

	macro-AUC			macro-F1		
CV-WBS	EE-WBS	RLWBS	CV-WBS	EE-WBS	RLWBS
LSTM	0.913	0.921	**0.923**	0.637	0.651	**0.682**
LSTM-bidir	0.917	0.923	**0.926**	0.642	0.677	**0.698**
ResNet	0.921	0.924	**0.928**	0.674	0.682	**0.707**
Inception	0.918	0.923	**0.927**	0.650	0.673	**0.701**
XResNet	0.923	0.926	**0.928**	0.683	0.700	**0.712**
LDM-XResNet	0.926	0.931	**0.935**	0.730	0.739	**0.762**

**Fig 6:**
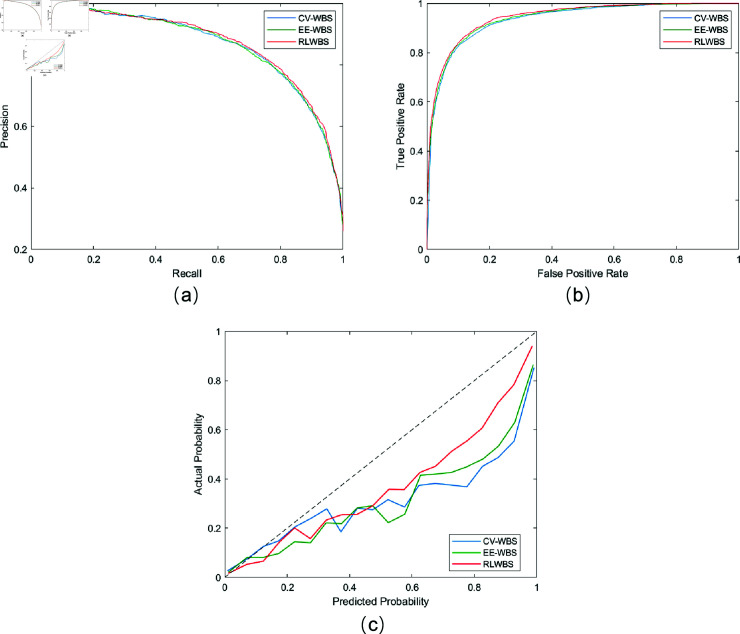
Different performance curves. (a), (b), and (c) are curves of precision-recall, ROC, and calibration, respectively.

Fig 6 presents various performance curves, including precision-recall, ROC, and calibration curves with the three WBS methods. Using the identical state-of-the-art (SOTA) classifier, the precision-recall and ROC curves achieved with RLWBS consistently outperform those achieved with CV-WBS and EE-WBS, demonstrating superior performance with RLWBS. Furthermore, as observed from the calibration curves, all three classifiers with the respective WBS mechanisms exhibit a tendency to be overconfident in their predictions, where the predicted probabilities are higher than the actual probabilities. Nevertheless, the calibration curve obtained with RLWBS aligns more closely with the ideal calibration curve compared to the others, indicating higher effectiveness and reliability of the proposed method.

These outcomes highlight the advantage of adaptive wavelet selection in our RLWBS method over static approaches. The CV-WBS method, which selects a single wavelet base that performs best on average, may overlook unique features in subsets of ECG signals that deviate from the majority, resulting in suboptimal performance for some cases. The EE-WBS approach, which selects a wavelet base based on morphological similarity to ECG signals, considers only one aspect of wavelet characteristics, potentially missing other factors such as support and vanishing moments. In contrast, the RLWBS framework enables the policy network to iteratively optimize WBS by maximizing classification accuracy through reward-based feedback from previous iterations. This continuous learning process enhances the capability of the network to select increasingly effective wavelet bases, while the adaptive nature of RLWBS ensures that extracted features align closely with the specific characteristics of each ECG signal. This adaptability could contribute to more effective feature extraction and improved classification performance in ECG diagnosis.

Additionally, Table 3 provides a detailed comparison of precision, recall, specificity, F1, and MCC scores achieved by LDM-XResNet using different WBS methods across all the diagnostic categories. The proposed RLWBS method consistently achieves higher precision and recall, indicating its stronger ability in identifying both positive and negative cases, thereby reducing both missed detections and false alarms. Hence, the proposed RLWBS could strike a better balance between false positives and false negatives, resulting in improved F1 scores. Moreover, the proposed method demonstrates superior performance in specificity and MCC, which indicates a lower misdiagnosis rate and a higher correlation between the predictions and the ground truth. These results further validate the efficacy of the proposed method in selecting the more appropriate wavelet base, ensuring more accurate and reliable ECG classification.

**Table 3:** Category-wise performance comparison of different WBS methods.

Precision	CD	HYP	MI	NORM	STTC	macro-	micro-
CV-WBS	0.775	0.615	0.757	0.763	0.772	0.736	0.774
EE-WBS	0.782	0.613	0.742	0.751	0.751	0.730	0.765
RLWBS	0.786	0.642	0.774	0.784	0.757	0.749	0.783
**Recall**							
CV-WBS	0.745	0.451	0.765	0.902	0.760	0.725	0.792
EE-WBS	0.732	0.566	0.782	0.917	0.742	0.748	0.802
RLWBS	0.755	0.603	0.795	0.933	0.798	0.777	0.821
**Specificity**							
CV-WBS	0.810	0.496	0.831	0.959	0.809	0.781	0.865
EE-WBS	0.803	0.600	0.836	0.983	0.796	0.804	0.927
RLWBS	0.819	0.648	0.842	0.976	0.865	0.830	0.948
**F1**							
CV-WBS	0.769	0.541	0.761	0.827	0.766	0.730	0.783
EE-WBS	0.756	0.589	0.761	0.826	0.751	0.739	0.783
RLWBS	0.770	0.622	0.784	0.852	0.777	0.762	0.802
**MCC**							
CV-WBS	0.556	-0.05	0.595	0.802	0.569	0.494	0.560
EE-WBS	0.536	0.166	0.612	0.819	0.539	0.535	0.655
RLWBS	0.576	0.251	0.635	0.841	0.655	0.591	0.665

Furthermore, we compare the SOTA model, LDM-ResNet1d, which originally uses 1D ECG signals, to its modified version adapted for wavelet feature input, i.e., RLWBS+LDM-XResNet2d in Table 4. When combined with RLWBS, the modified LDM-ResNet model achieves even higher macro-F1 scores. This improvement further demonstrates the efficacy of the proposed RLWBS framework in enhancing the inference capacity of DL models for ECG diagnosis.

**Table 4:** Comparison of F1 scores between the original LDM-XResNet1d and the modified LDM-XResNet2d model with RLWBS.

	CD	HYP	MI	NORM	STTC	macro-F1
LDM-XResNet1d [40]	0.763	0.571	0.767	0.862	0.747	0.742
RLWBS+LDM-XResNet2d	0.770	0.622	0.784	0.852	0.777	0.762

### Comparison of extracted wavelet features

**Fig 7:**
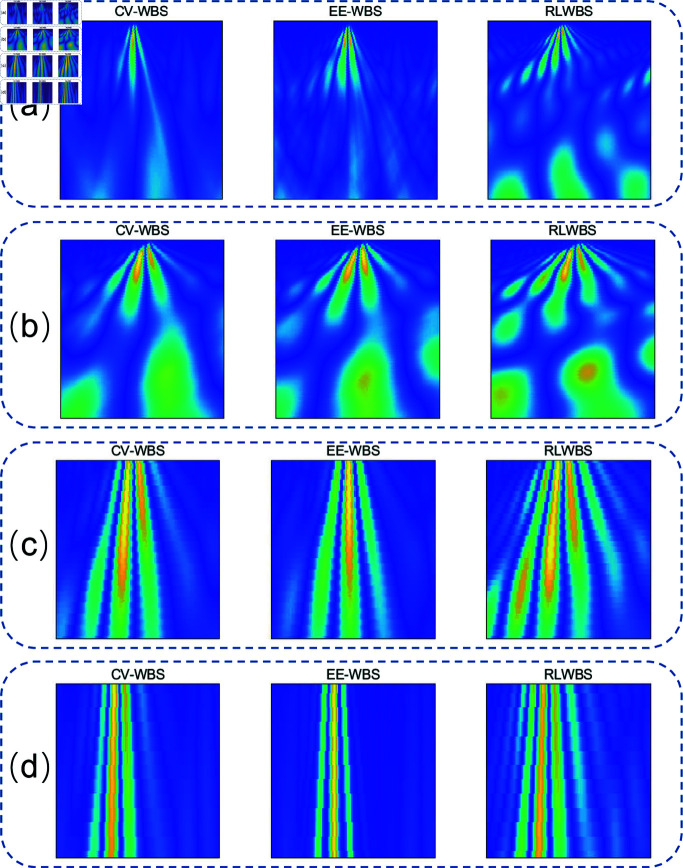
Examples of extracted features generated by different wavelet selection methods across varying time-frequency scales. (a), (b), (c), and (d) focus on the same time-frequency regions, respectively.

To assess the effectiveness of wavelet bases selected by the RLWBS framework for feature extraction, we present examples of wavelet features generated by each of the three WBS methods in Fig 7. These scalograms, produced through CWT of the same ECG signal segments, demonstrate that the time-frequency information obtained via the RLWBS framework captures finer detail and variation than the other methods, particularly in areas with rapid and complex frequency changes. Hence, it indicates that the RLWBS method provides greater detail in the time-frequency representation, effectively capturing the diversity of frequency components.

Unlike many existing approaches [19, 33, 41, 42], which predetermine wavelet bases during the preparation stage, the RLWBS framework adaptively selects wavelet bases, providing a higher degree of freedom in capturing time-frequency features. By adjusting wavelet wavelet base to align with the unique characteristics of different signals, RLWBS produces a clearer, more accurate depiction of signal time-frequency dynamics. This adaptability yields more detailed and relevant time-frequency information according to categories, which is particularly advantageous for analyzing complex signals, as it captures subtle variations and potential anomalies more effectively.

### Analysis of selected wavelet bases by RLWBS

Table 5 presents the distribution of wavelet base families selected by both the EE-WBS and RLWBS methods. Notably, neither method selects the Haar wavelet, as its discontinuous nature makes it unsuitable for capturing ECG signal features. Additionally, both methods share a similar distribution pattern, with the db wavelet family chosen most frequently, followed by the sym, bior, and coif wavelet families. This pattern reflects the significance of morphological similarity between the ECG signal and wavelet bases, which is the core idea followed by EE-WBS. However, while both approaches emphasize similarity, RLWBS seems to extend beyond this criterion by integrating additional factors influencing wavelet selection compared to EE-WBS. By training a policy network guided by classification performance, RLWBS adapts dynamically, optimizing wavelet selection not only based on similarity but also on other critical factors that enhance feature extraction. This adaptability results in a more refined and effective wavelet-based feature extraction process, particularly valuable for ECG diagnosis.

**Table 5:** Distribution of selected wavelet base families with EE-WBS and RLWBS.

	haar	db	sym	bior	coif
EE-WBS	0%	45%	37%	14%	4%
RLWBS	0%	53%	38%	7%	2%

**Fig 8:**
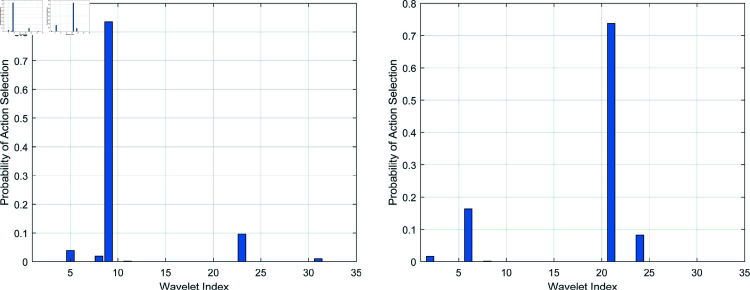
Two examples of the probability distribution output from the policy network.

Fig 8 illustrates two examples of the action probability distributions generated by the policy network for two distinct ECG signals. The high certainty in selecting specific actions upon training completion demonstrates the convergence of action learning, as the policy network identifies a wavelet base for each input signal with high confidence. It suggests that the network successfully detects unique patterns or features within each signal, enabling further decision-making accordingly. Furthermore, the adaptivity of the policy network can be observed as it dynamically selects wavelet bases tailored to different ECG signals. This adaptive approach aims to optimize wavelet selection on a per-signal basis, ultimately enhancing classification performance by aligning the wavelet base more closely to the characteristics of each signal.

### Model interpretability through GradCAM

**Fig 9:**
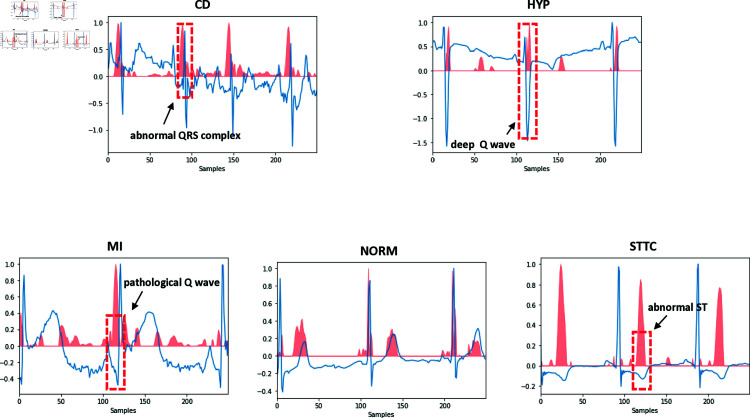
GradCAM for different categories.

Fig 9 illustrates the focus of the model trained with RLWBS on the ECG signals, represented by the blue curves, while the red curves depict the attention values generated by the classifier. For abnormal categories, the model assigns high attention values to the abnormal ECG regions, effectively highlighting diagnostically relevant segments. Additionally, critical regions of normal ECG signals are carefully examined to confirm the absence of abnormal features. This highlights the capability of the classifier with RLWBS to capture distinctive features pertinent to cardiac diseases, aligning with clinical considerations and providing valuable support to healthcare practitioners in diagnosis.

### Limitations and future works

Compared to traditional WBS approaches, such as EE-WBS, which determine the appropriate wavelet base solely by measuring the correlation between ECG signals and wavelet bases, the proposed method requires a training dataset with fully annotated labels. These labels provide performance guidance, enabling the policy network to refine its policy generation strategy. Additionally, the efficacy of the proposed method may degrade with smaller training datasets, as training of the policy maker for WBS heavily rely on the amount of data available for training. This dependency on large volumes of annotated data could be a limitation in scenarios where data is limited, such as when data is streaming or labeled datasets are scarce or expensive to obtain.

A potential solution to these limitations could be transfer learning [43]. Specifically, the knowledge gained from an agent trained on one dataset could be transferred to other ECG signals or different application domains with distinct classification categories, thus enabling broader applicability. To tackle the challenge of unlabeled datasets, unsupervised learning techniques [44] could be employed to adjust the policy maker online, even with limited data annotations. This would help reduce the reliance of training policy maker on fully labeled datasets and extend the usability of the proposed method in real-world scenarios where labels are not readily available. Additionally, in situations with limited data samples, few-shot learning [45] could be utilized to enable the trained agent to adapt its action generation with minimal training samples, effectively mitigating data scarcity and further enhancing the adaptability of the proposed approach.

## Conclusion

This study introduces an RL-based WBS mechanism designed to enhance classification performance in DL-based ECG diagnosis. The approach enables an RL agent to dynamically select the optimal wavelet base for each ECG signal, matching its unique characteristics and thus providing more informative wavelet features for improved classification performance. Specifically, the WBS task is framed as an MDP and solved through a PG method, with particularly designed configurations for state, action, and reward. Performance evaluation with the PTB-XL dataset shows that, unlike traditional methods, which often rely on static wavelet bases selected through trial-and-error or similarity to ECG signal morphology, the proposed RLWBS framework achieves finer ECG feature capture, yielding higher level of classification outcomes. It highlights the potential of RL-driven wavelet selection to advance DL-based ECG diagnostic models.
